# Relationship between gait parameter and spinal sagittal profiles in asymptomatic subjects

**DOI:** 10.1186/s12891-023-06672-8

**Published:** 2023-07-08

**Authors:** Sirichai Wilartratsami, Lopburi Nathasiri, Bavornrat Vanadurongwan, Borriwat Santipas, Siravich Suvithayasiri, Panya Luksanapruksa

**Affiliations:** 1 Department of Orthopaedic Surgery, Faculty of Medicine Siriraj Hospital, Mahidol University, 2 Wanglang Road, Bangkok, 10700 Thailand; 2 Orthopedic Center, Chulabhorn Hospital, Chulabhorn Royal Academy, Bangkok, Thailand

**Keywords:** Gait analysis, Pelvic incidence, Gait parameters, Asymptomatic volunteer, Sagittal profiles

## Abstract

**Background:**

To study the gait parameters in asymptomatic volunteers and investigate the correlation between the gait and several radiographic sagittal profiles.

**Methods:**

Asymptomatic volunteers (20–50 years of age) were included and allocated into three subgroups depending on pelvic incidence (low, normal, and high). Standing whole spine radiographs and gait analysis data were obtained. The Pearson Coefficient Correlation was used to determine the relationship between the gait and radiographic profiles.

**Results:**

A total of 55 volunteers (28 male and 27 females) were included. The mean age was 27.35 ± 6.37 years old. The average sacral slope (SS), pelvic tilt (PT), pelvic incidence (PI), and PI-LL mismatch (PI-LL) were 37.78 ± 6.59, 14.51 ± 9.19 degrees, and 52.29 ± 10.87 degrees and − 0.36 ± 11.41, respectively. The mean velocity and stride of all the volunteers were 119.00 ± 30.12 cm/s and 130.25 ± 7.72 cm, correspondingly. The correlation between each of the radiographical and gait parameters was low (ranging from − 0.24 to 0.26).

**Conclusion:**

Gait parameters were not differenced significantly between each of the PI subgroups in asymptomatic volunteers. Spinal sagittal parameters also showed a low correlation with gait parameters.

## Background

Adult spinal deformity (ASD) often has a serious impact on a patient’s health-related quality of life (HRQoL), with a prevalence of up to 60% in the elderly population [1–3]. Progression of pain and function disabilities, severe deformity, and/or neurological compromise are indicated for surgical treatment [4]. The main goal is to restore the patient’s sagittal balance [5]. Therefore, appropriate information on physiologic pelvic parameters and sagittal balance is crucial when treating ASD [6, 7]. Moreover, deviation in one’s sagittal profiles may further cause a disturbance in the gait of the patients and, consequently, cause changes in its parameters [8–10]. Many studies have already reported sagittal profile parameters in asymptomatic volunteers [11–19], but few have established its relationship with the gait [20, 21]. Patients with spinal deformities have been found to exhibit significant alterations in these parameters, which are strongly associated with a decline in their quality of life. Despite this, the impact of these postural parameters on gait, even in asymptomatic adults, remains unclear. Therefore, the objective of this study was to investigate the correlation between several radiographic sagittal profiles and gait kinematics in asymptomatic adults. We hypothesized that there might be some relationships between the two concerning profiles, even among those asymptomatic subjects.

## Methods

After obtaining approval from our Institutional Review Board (IRB) under at Siriraj Hospital. The IRB number 435/2560(EC1). All participants or their legal guardian signed an informed consent for publication of identifying information in an online open-access publication. we enrolled volunteers who met the following inclusion criteria: aged between 20 and 50 years old, with no spinal deformities or abnormalities on radiographic studies (Cobb angle less than 20 degrees in both coronal planes and no deformity in sagittal planes). The exclusion criteria were as follows: inability to walk dependently, previous joint replacement or spinal surgery, prior gait problems, inappropriate radiographic studies, neuromuscular conditions that could affect gait, and those with contraindications for radiographic examination, such as pregnancy. Moreover, individuals with a prior history of trauma, ischemic, or any other vascular pathologies of the lower limbs were also excluded. All participant data were collected at our institution during Jan-Dec 2019. Participants were received the radiological examination and gait analysis. The collected data were shown below, in the Radiographic measurement and gait analysis section.

### Radiographic measurement

All those volunteers were undergoing a standard plain radiograph of the whole Spine, including an anteroposterior (AP) and lateral (Lat) view in a standing position. Digital images of the whole spine were reconstructed (stitched) and evaluated by software application of picture archiving and communication system (Synapse PACS HL7 Interface version 5, FUJIFILM MEDICAL SYSTEMS, U.S.A.).The sagittal profile parameters were measured following the previous studies of Morvan et al. [22], including coronal vertical axis (CVA), C7 sagittal vertical axis (C7SVA), pelvic incidence (PI), sacral slope (SS), pelvic tilt [4], lumbar lordosis (LL), PI-LL mismatch, thoracic kyphosis (TK), thoracolumbar kyphosis (TLK), T1 spinopelvic inclination (T1Spi), and the T1 pelvic angle (TPA).

In the AP view, CVA was measured as a distance between the C7 plumb line and a point at the middle of the sacral endplate. Then, on the lateral view radiograph, C7SVA was measured as the distance between the vertical plumb line and the point at the superior-posterior border of the sacral endplate. PI was measured as an angle between the line perpendicular to the sacral endplate and the line drawn from the middle point of that sacral endplate and the middle point of the line that connects the center of the two femoral heads (bicoxofemoral axis). SS was measured as an angle between the horizontal plane and a line parallel to the sacral endplate. PT was measured as an angle between the vertical plane and the line drawn from the middle point of the sacral endplate to the middle point of the bicoxofemoral axis. LL is defined as an angle formed by the line parallel to the upper endplate of the T12 vertebral body and the line parallel to the sacral endplate (upper endplate of the S1 level). The same technique was applied when measuring the TK (T5-T12) and TLK (T10-L2). The T1Spi is defined as the angle between the vertical plumb line and the line drawn from the vertebral body centroid of T1. Lastly, TPA is defined as the angle between the lines from the center of the bicoxofemoral axis to the middle point of the sacral endplate. The volunteers were then divided into three groups as related to their PI value.

According to Labelle et al. [23], The volunteers were classified into three groups based on PI value. The low PI (LPI), Normal PI (NPI), and the High PI (HPI) group have a PI value of < 45 degrees, 45–60 degrees, and > 62 degrees, respectively.

### Gait analysis

**Fig. 1:**
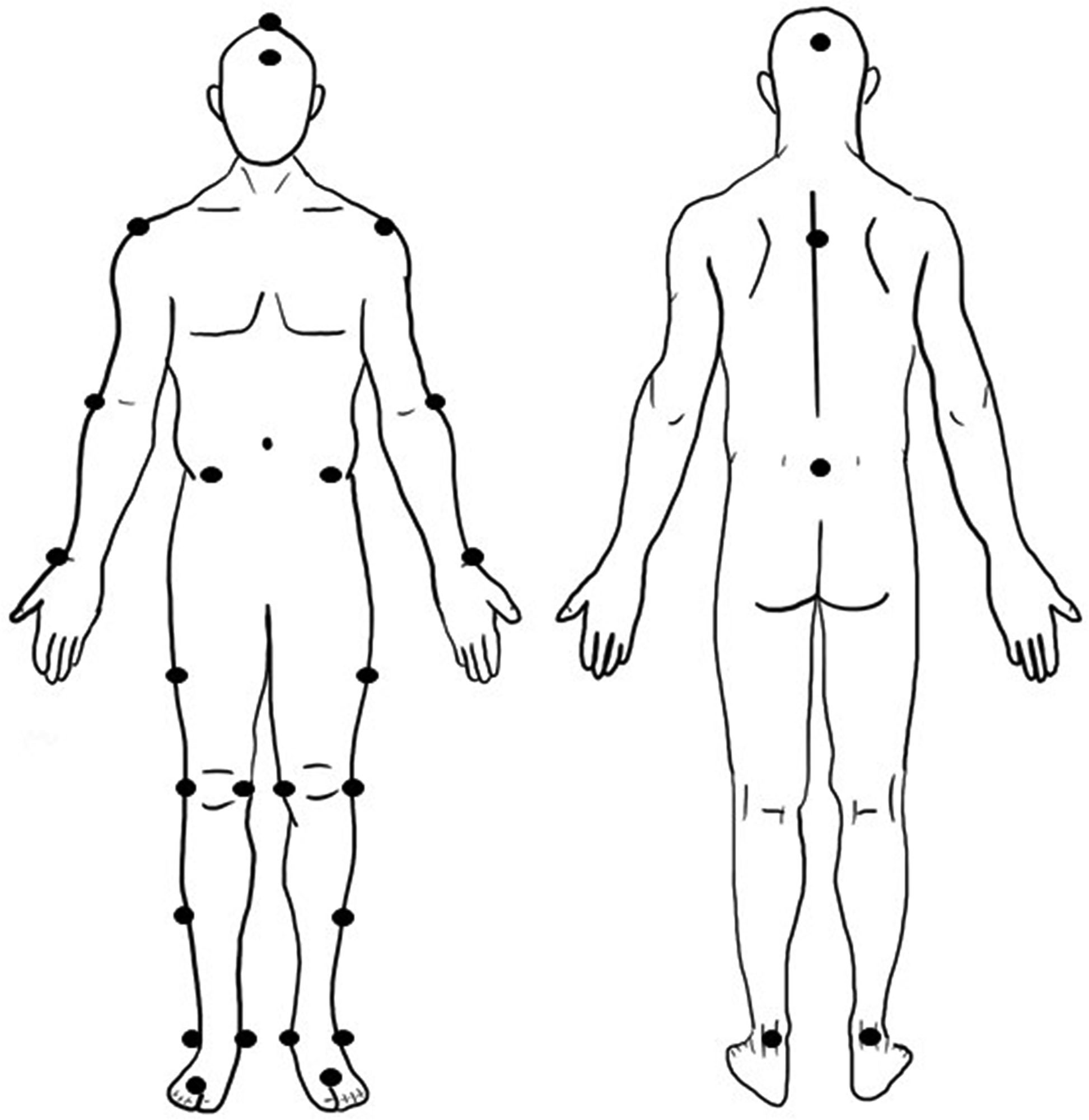
The Helen Hayes Model – 29 reflective markers were used to attach to each volunteer before the trial according to the Helen Hayes model. This is the diagram figure draw by author

Using the Helen Hayes model [24], 29 reflective markers were attached to the volunteer bony landmarks. (Fig. 1.) An eight-camera motion analysis system (Motion Analysis Corp., Santa Rosa, CA) will synchronize with 2 force plates (ATMI, Watertown, MA). Then, the volunteer would be asked to stand on the center of the volume for the static trial to determine his or her axis of rotation and joint center. Afterward, he or she would walk at a self-selected speed on a 10-meter walkway three times. Gait parameters were recorded, including velocity, stride, cadence, step length, and percentage of each phase of the gait cycle (e.g., stance, double stance, and swing phase).

### Statistical analysis

To define the relationship between gait and radiographical profiles, the Pearson Coefficient Correlation was used if the variable was linear. The one-way analysis ANOVA was used to find an association between gait and each PI subgroup. If the result was statistically significant, then the *post hoc* analysis with the Bonferroni method, or the Kruskal-Wallis analysis would be used in the normal distribution data, and the abnormal distribution data, respectively. A *p*-value of less than 0.05 with a confidence interval of 95% was considered statistically significant. All the analyses were done using the SPSS Statistics version 18.0 (IBM Corporation, SPSS Inc., Chicago, IL, USA).

## Results

A total of 28 male and 27 females were included. The mean age was 27.35 years (range, 20–44). Age, weight, and BMI showed significant differences between groups (P-values = 0.02, 0.01, and 0.01, respectively). Other demographic data, including height, weight, and body mass index (BMI) are also shown in Table 1.

**Table 1:** – Demographic data and comparison between the PI subgroups

	All group	Low PI	Normal PI	High PI	p-value
Number	55	10	34	11	0.10
Female	27	8	16	4	
Male	28	2	18	7	
Age (years)	27.35 ± 6.37	24.40 ± 4.03	28.38 ± 6.66	26.82 ± 6.75	0.02*
Height (cm)	166.65 ± 6.90	165.90 ± 5.13	165.88 ± 7.34	169.73 ± 6.51	0.26
Weight (Kg)	62.84 ± 12.46	55.55 ± 8.78	62.32 ± 10.48	71.09 ± 16.65	0.01*
BMI (Kg/m^2^)	22.53 ± 3.72	20.12 ± 2.50	22.55 ± 2.75	24.69 ± 5.78	0.01*

* = statistically significant. PI: pelvic incidence, BMI: body mass index

The detail radiographic measurement is revealed in Table 2. Considering the sagittal profiles, because we have divided the volunteer depend on his or her PI and because of the relationship between PI and other several parameters, the mean value of SS, PT, LL, PI, PI-LL, and TPA between the three subgroups are shown to be statistically significant difference. In contrast to the results observed for spinopelvic angles, the gait parameters exhibited different behavior. Regression for mean was employed to compare the primary outcome between the PI subgroups, adjusted for potential confounders such as age and BMI. All measured gait parameters, including the percentage of each phase of the gait cycle, were found to be relatively the same without any increasing or decreasing trend of value compared to the PI. All of them were not statistically significant difference. The detail gait analysis results is shown in Table 3.

**Table 2:** Radiographic sagittal profiles and comparison between the PI subgroups

	Total	Low PI	Normal PI	High PI	p-value
CVA (cm)	-0.50 ± 0.92	-0.42 ± 0.93	-0.58 ± 0.90	-0.33 ± 1.03	0.715
C7SVA (cm)	-0.76 ± 2.34	-2.23 ± 2.38	-0.93 ± 1.88	1.11 ± 2.60	0.004*
TK (°)	26.91 ± 9.63	26.20 ± 7.08	27.74 ± 9.35	25.00 ± 12.68	0.571
TLK (°)	5.89 ± 9.44	8.90 ± 6.28	5.32 ± 9.59	4.91 ± 11.44	0.542
LL (°)	52.65 ± 8.57	47.20 ± 9.35	53.00 ± 7.49	56.55 ± 9.28	0.038*
SS (°)	37.78 ± 6.59	31.30 ± 5.54	38.12 ± 5.60	42.64 ± 5.92	< 0.001*
PT (°)	14.51 ± 9.19	5.90 ± 8.09	13.12 ± 5.88	26.64 ± 6.39	< 0.001*
PI (°)	52.29 ± 10.87	37.20 ± 4.57	51.24 ± 3.93	69.27 ± 4.47	< 0.001*
PI-LL (°)	-0.36 ± 11.41	-10.00 ± 12.77	-1.76 ± 7.27	12.73 ± 9.70	< 0.001*
T1Spi (°)	-5.27 ± 1.98	-5.30 ± 2.50	-5.12 ± 1.77	-5.73 ± 2.20	0.681
TPA (°)	9.24 ± 8.84	0.60 ± 7.23	8.00 ± 5.67	20.91 ± 6.25	< 0.001*

* = statistically significant. PI: pelvic incidence, CVA: coronal vertical axis, C7SVA: C7 sagittal vertical axis, TK: thoracic kyphosis, TLK: thoracolumbar kyphosis, LL: lumbar lordosis, SS: sacral slope, PT: pelvic tilt, PI-LL: PI-LL mismatch, T1Spi: T1 spinopelvic inclination, TPA: T1 pelvic angle

**Table 3:** Gait parameters and comparison between the PI subgroups

	Total	Low PI	Normal PI	High PI	p-value*
Velocity (cm/s)	119.00 ± 30.12	129.69 ± 10.06	117.48 ± 31.08	125.58 ± 8.65	0.692
Right stride (cm)	129.60 ± 7.85	130.75 ± 5.9	129.57 ± 8.31	131.47 ± 7.37	0.935
Left stride (cm)	129.76 ± 7.98	130.84 ± 5.76	129.82 ± 8.47	131.47 ± 7.69	0.966
Step length (cm)	65.10 ± 3.84	65.43 ± 3.02	64.80 ± 4.16	65.72 ± 3.67	0.936
Cadence (steps/min)	115.58 ± 6.74	118.66 ± 7.39	115.02 ± 6.30	114.43 ± 7.31	0.390
Stance phase (%)	59.60 ± 1.05	59.34 ± 1.56	59.57 ± 0.88	59.91 ± 1.02	0.459
Swing phase (%)	40.40 ± 1.05	40.66 ± 1.56	40.43 ± 0.88	40.09 ± 1.02	0.459
Double stance phase (%)	9.50 ± 1.10	9.21 ± 1.64	9.52 ± 0.92	9.87 ± 1.01	0.531

*Age and BMI adjusted regression analysis

As displayed on Table 4., the correlation coefficient between radiographic sagittal profiles and gait parameters, none was found to be difference statistical significantly. The Pearson correlation coefficient is ranged from − 0.24 to 0.26. This result show low to very low correlation between each parameter of the gait and sagittal profile.

**Table 4:** Correlation coefficient between sagittal profiles and gait parameters

	Velocity	Stride	Cadence	Stance phase	Swing phase	Double stance phase
C7SVA (cm)	-0.06	0.04	-0.11	0.13	-0.13	0.16
SS (°)	0.1	0.15	-0.03	0.14	-0.14	0.15
PT (°)	-0.22	-0.14	-0.15	0.18	-0.18	0.21
PI (°)	-0.13	-0.03	-0.14	0.24	-0.24	0.26
PI-LL (°)	-0.2	-0.08	-0.17	0.24	-0.24	0.26
TK (°)	-0.09	-0.03	-0.09	0.01	-0.01	0.004
LL (°)	0.09	0.07	0.04	-0.01	0.01	-0.01

Abbreviation - PI: pelvic incidence, C7SVA: C7 sagittal vertical axis, TK: thoracic kyphosis, LL: lumbar lordosis, SS: sacral slope, PT: pelvic tilt, PI-LL: PI-LL mismatch

## Discussion

Differences in gait between individuals are influenced by several factors. According to a study conducted by Oberg et al., younger males had a significantly faster gait, longer stride, and lower step frequency than older females [25]. Additionally, variations were observed when comparing gait patterns between different races and ethnicities. When German and Asian volunteers were compared, the latter tended to walk slower with shorter strides, although the percentage of the stance, swing, or double stance phase was relatively similar [26, 27]. Interestingly, the gait velocity was found to differ between volunteers from Germany and Sweden [25]. These findings suggest that several factors, including physical factors such as anatomical or physiological differences and non-physical factors such as ethnicity, race, and culture, can contribute to gait. In our study, we found that volunteers tended to have a slower gait speed compared to the German study, but similar to the other studies. Moreover, although the stride was relatively close to the study by Al-Obaidi et al. [28], it was lower than that of the German volunteers, but higher than those of Swedish and Japanese volunteers.

There are few studies that have addressed the relationship between sagittal profiles and gait in symptomatic ASD patients. Engberg et al. compared prospectively between preoperative and postoperative gait of 29 revision or primary patients having long fusions from thoracic to the distal lumbar Spine or sacrum level. Improvement in gait endurance and speed was shown but not significantly different 2 years after surgery [29]. However, Gottipati et al. reported that after the multi-segment reconstructive spine correction surgery, it could either resolve a crouching posture and gait or enhance sagittal spinal alignment and improve the step length significantly [20]. Fewer studies have been reported about this relationship in asymptomatic subjects. A prospective case series by Yagi et al. [27], assessed the gait pattern in patients with adult spinal deformity (ASD) and compared them with age- and gender-matched health volunteers. After surgery, the gait pattern, stride, and velocity improved significantly in ASD patients. There is a moderate correlation between the SRS22 satisfaction domain and postoperative gait velocity. Noteworthy, low to moderate correlations were found between several preoperative sagittal profiles and gait kinematics, both positive and negative directions (*r* range from − 0.72 to 0.61). Unfortunately, the authors did not statistically analyze the correlation coefficients between sagittal profiles and gait parameters in asymptomatic subjects, so head-to-head comparison with our study is impossible.

A previous study by Otayek et al.[30], investigated the influence of spino-pelvic and postural alignment parameters on gait kinematics in asymptomatic subjects. The study showed that increasing sagittal vertical axis, center of auditory meatus to hip axis plumbline, thoracic kyphosis, and radiologic pelvic tilt, which are known to occur in adults with spinal deformities, could alter gait kinematics. Specifically, the study found a decrease in walking pace, reduced speed, shorter step length, and a longer stance phase.

Our study provides the relationship between sagittal profiles and gait parameters in 55 asymptomatic individuals. The results of our study are compatible with those of previous studies in different races or ethnicities, except for the pelvic incidence (PI) and thoracic kyphosis (TK) [16, 31–33]. In the present study, gait parameters were not differenced significantly between each of the PI subgroups in asymptomatic volunteers. Spinal sagittal parameters also showed a low correlation with gait parameters. Although gait parameters were not differenced significantly between each of the PI subgroups, the Pearson’s correlation coefficient between sagittal profiles and the gait parameters is valid in either positive or negative directions (*r* range from − 0.24 to 0.26). Regarding each sagittal parameter, the correlation coefficient between PT and gait velocity, stride, and cadence were all negative. This could be due to the increase in PT (with a decrease in SS), which may have increased pelvic retroversion during gait, and thus require more maximal hip extension during stance, ultimately affecting gait ability. The SVA, which also known as one of the strongest correlations with quality of life in adult spinal deformity patient, had the negative correlation with the velocity, swing phase and posititve correlation with the stance phase and double stance phase. An increase in SVA is often associated with a forward shift of the body’s center of mass, which can cause an individual to lean forward while walking. To compensate for this forward shift, individuals with an increased SVA may take shorter steps and have a longer stance phase, which refers to the period of time when the foot is in contact with the ground [34]. Additionally, an increased SVA can lead to a decrease in the ability of the hip extensor muscles to generate the necessary force to propel the body forward during walking. This decrease in hip extensor function can lead to a longer double stance phase, which is the period of time when both feet are in contact with the ground during the gait cycle. Of course, this study was conducted in asymptomatic subjects, and many factors would come into play to compensate for this problem (e.g., increased the sagittal plane range of motion of the ankle, muscle strength, etc.) – thus, resulting in a low correlation. However, with low correlation, asides from the sagittal profiles, it does not necessarily mean that the gait ability alone does not directly affect the clinical outcomes of the patients with ASD. Future studies to directly investigate the relationship between gait parameters and functional status in these patients could give us a wider view and better understanding of this gap of knowledge, much or less.

There are some limitations to our study. First, a small sample size could have limited our ability to draw better conclusions from these findings. Also, because of the small sample size, the unequal gender and age distribution among the subgroups that might affect the outcome of this study could not be addressed appropriately. Second, because of the cross-sectional nature of this study, a longitudinal study that could compare each parameter according to their age and various sagittal profiles could be better if possible. Moreover, without the degenerative changes in their Spine, the young asymptomatic population would usually still have a proportion of alignment of their Spine to be good. Thus, the effect on their gait would be none or shown only a little compared to those of older ages. Future studies should also focus on comparing the impact on gait between those with asymptomatic and symptomatic ASD. Asides from these limitations, to the best of our knowledge, this is the first study to demonstrate a relationship between each of the variables of sagittal profiles and gait parameters.

## Conclusions

our study provides valuable insights into the relationship between sagittal profiles and gait parameters in asymptomatic individuals. Although the correlation coefficient between sagittal profiles and gait parameters is low, it suggests that many factors come into play to compensate for this problem in asymptomatic individuals. Future studies should focus on comparing the impact on gait between those with asymptomatic and symptomatic ASD.

## Data Availability

The data used to support the findings of this study are available from the corresponding author upon request.

## List of Abbreviations

ASD: Adult spinal deformity
HRQoL: Health-related quality of life
IRB: Institutional Review Board
AP: Anteroposterior
Lat: Lateral
CVA: Coronal vertical axis
C7SVA: C7 sagittal vertical axis
PI: Pelvic incidence
SS: Sacral slope
PT: Pelvic tilt
LL: Lumbar lordosis
TK: Thoracic kyphosis
TLK: Thoracolumbar kyphosis
T1Spi: T1 spinopelvic inclination
TPA: T1 pelvic angle
LPI: Low PI
NPI: Normal PI
HPI: High PI
BMI: Body mass index
